# Die zahnärztliche Früherkennungsuntersuchung – eine systematische Analyse des Informationsgehalts nationaler Kinderzahnarztpässe

**DOI:** 10.1055/a-2573-5692

**Published:** 2025-04-30

**Authors:** Kimija Rahbari, Christian Graetz, Miriam Cyris, Malin Sucherlan, Christof E. Dörfer, Antje Geiken

**Affiliations:** 1 Klinik für Zahnerhaltungskunde und Parodontologie, Universitätsklinikum Schleswig-Holstein, Campus Kiel, Kiel, Germany

**Keywords:** Zahnärztliche Frühuntersuchungen, Fluoride, Kariesprävention, Kinder, Gesundheitsfürsorge, early dental visit, fluorides, caries prevention, children, healthcare

## Abstract

**Ziel:**

Zahnärztliche (ZA) Kinderpässe (ZKP) – als Pendant zu den Kinderuntersuchungsheften – haben einen informellen Charakter sowie eine Erinnerungsfunktion für die Früherkennungsuntersuchungen (FU1a-c, FUPr, FLA). So soll langfristig eine Prävention vom Neugeborenen bis zum 6. Lebensjahr erfolgen. Jedoch haben die 15 zahnärztlichen Kammerbereiche (KB) teils eigene ZKPs (n=14), sodass die inhaltliche und formale Evaluation dieser ZKPs das Ziel der Untersuchung war.

**Methode:**

Vierzehn ZKPs wurden von 12 Zahnärzten mit verschiedenem Tätigkeitsschwerpunkt (je 2 Kinderzahnmedizin, Parodontologie, Prothetik, Zahnerhaltung, Kieferorthopädie, keine Spezialisierung) vom 01.07.2023–30.11.2023 anhand eines validierten Kriterienkataloges (81 Items) bewertet, bestehend aus Fragen zur Qualität (Wittener Liste, DISCERN, Hamburger Verständlichkeitsmodell) sowie bezüglich des Inhalts der ZKPs. Neben einer deskriptiven Analyse erfolgte die statistische Auswertung mittels des Kruskal-Wallis-Tests, eines asymptomatischen Signifikanztests, einer Korrelationsanalyse der Qualität für Patienteninformationen (Spearman-Korrelation), einer binären logistischen Regressionsanalyse der Variablen Untersucherspezialisierung (auf Kinderzahnmedizin/nicht auf Kinderzahnmedizin), Untersuchergeschlecht (weiblich/männlich), Untersucherberufserfahrung (≤7Jahre/>7Jahre) und KB (östlicher/westlicher Kammerbereich) des ZKPs auf den inhaltlichen Summenwert des ZKPs.

**Ergebnisse:**

Der ZKP des KBs Bremen/Schleswig-Holstein (identischer ZKP) war inhaltlich am besten bewertet mit einem Median (25%/75% Perzentil) von 100,50(100,00/101,75), der ZKP Brandenburg am schlechtesten 69,00(66,50/73,00); p>1,00). DISCERN und das Hamburger Verständlichkeitsmodell korrelierten (ρ=0,565 (p<0,001)). Die Regressionsanalyse zeigte nur für den KB des ZKPs einen signifikanten Einfluss auf die inhaltliche Bewertung (p<0,001).

**Schlussfolgerung:**

Eine starke Diskrepanz in der Qualität sowie inhaltlichen Quantität der ZKPs war vorherrschend. Eine einheitliche Form und inhaltliche Kongruenz der ZKPs, wie bereits für die Kinderuntersuchungshefte bestehend, ist anzustreben.

## Abstract (en)

**Aim:**

Dental child health records have an informal character and act as a reminder of early dental visits (FU1a-c, FUPr, FLA). Long-term prevention should take place from newborns to the age of 6. However, each chamber area (n=17) has its own dental child health record (n=14). The evaluation of the formalities and the content was the aim of this study.

**Method:**

Fourteen dental child health records were evaluated by 12 dentists with different specializations (2 each in pediatric dentistry, periodontics, prosthetics, tooth preservation, orthodontics, no specialization) from July 1st, 2023 - November 30th, 2023. The dentists used a catalogue of criteria with 81 items. The validated criteria catalogue consisted of questions about quality (Witten List, DISCERN, Hamburg Model of Comprehensibility) and other relevant topics. A descriptive analysis and statistical evaluations (Kruskal-Wallis test, asymptomatic significance test), a correlation analysis of the quality of patient information (Spearman correlation), a binary logistic regression analysis of the variables specialization of the examiner (in pediatric dentistry/not in pediatric dentistry), gender (female/male), professional experience (≤7 years/>7 years) and chamber area (eastern/western chamber areas) were carried out.

**Results:**

The dental child health record from Bremen/Schleswig-Holstein (identical) was rated best in terms of content median (25%/75% percentile): 100.50(100.00/101.75), the dental child health record of Brandenburg was rated the worst 69, 00(66.50/73.00); p>1.00). There was good correlation between DISCERN and the Hamburg Model of Comprehensibility (ρ=0.565 (p<0.001)). According to the regression analysis, only the chamber area showed a significant influence on the content (p<0.001).

**Conclusion:**

There was a strong discrepancy in the quality and quantity of the dental child health records. It is important to strive towards a standardized form and congruence in content similar to that which already exists for the children’s medical examination booklets.

## Einleitung

Entsprechend einer UN-Konvention 1 hat jedes Kind das Recht auf eine bestmögliche Gesundheit sowie Zugang zur Gesundheitsversorgung. Dabei stellt gerade die Early Childhood Caries (ECC) – die frühkindliche Karies bei sechsjährigen Kindern in Europa mit einer Prävalenz von 20–90% eine Herausforderung dar, insbesondere durch die Polarisierung auf die Bevölkerungsanteile mit einem geringeren Bildungsgrad und geringerem Einkommen 2 . Eine verschleppte frühzeitige Kariesprävention kann gravierende Folge für die Lebensqualität des (Klein-)Kindes haben 3 .

Somit sind gerade die Informationen, wie das korrekte Alter für eine erste zahnärztliche Untersuchung oder aktuelle Empfehlungen zur Fluoridierung von immenser präventiver Bedeutung 4 . Teilweise konnten zwar einige der genannten Aspekte durch interdisziplinäre Bestrebungen gelöst werden, beispielweise im Rahmen des Netzwerks „Gesund ins Leben“ vom 29. April 2021, innerhalb dessen eine gemeinsame Fluoridempfehlung veröffentlicht wurde, welche unter anderem ab dem 1. Lebensjahr des Kindes eine fluoridhaltige Zahnpasta (1000 ppm Fluorid) in Reiskorngröße und ab dem 2. Lebensjahr in Erbsengröße beschreibt. Auch kann mit den am 01.07.2019 neu eingeführten zahnärztlichen Früherkennungsuntersuchungen (FU1a-c, FU2), den praktischen Unterweisungen der Mundhygienemaßnahmen (FUPr) sowie der Fluoridlackapplikationen (FLA) eine Frühprävention innerhalb der kassenzahnärztlichen Versorgung stattfinden. Zahnärztliche Kinderpässe (ZKP) könnten dabei als wichtige deutschlandweite einheitliche präventive Interventionen gefördert werden. Sie führen alle altersentsprechenden Frühuntersuchungen der Mundhöhle des Kindes auf, sodass Eltern daran erinnert werden, stellen daneben aber auch weitergehende Informationen zur Mundgesundheit bereit und teils lassen sich auch Befunde dokumentieren. Von den Autoren der vorliegenden Arbeit wird jedoch als problematisch angesehen, dass in jedem der 15 zahnärztlichen Kammerbereiche in Deutschland (Landeszahnärztekammer Baden-Württemberg (Version 2022), Bayerische Landeszahnärztekammer (Version 2021), Zahnärztekammer Berlin (Version 2022), Landeszahnärztekammer Brandenburg (Version 2012), Zahnärztekammer Bremen (Version 2021, Zahnärztekammer Hamburg (Version 2020), Landeszahnärztekammer Hessen (Version 2021), Zahnärztekammer Mecklenburg-Vorpommern (keine Angabe), Zahnärztekammer Niedersachsen Version 2021), Zahnärztekammer Nordrhein (Version 2022), Landeszahnärztekammer Rheinland-Pfalz (keine Version angegeben), Zahnärztekammer des Saarlandes (Version unbekannt), Landeszahnärztekammer Sachsen (Version 2020), Zahnärztekammer Sachsen-Anhalt (Version 2020), Zahnärztekammer Schleswig-Holstein Version 2021), Landeszahnärztekammer Thüringen (keine Angabe), Zahnärztekammer Westfalen-Lippe (Version 2021)) unterschiedliche ZKPs veröffentlicht werden. Auch gibt es keinerlei allgemeine und nationale Richtlinien beziehungsweise Empfehlungen, wie medizinische/ präventive Informationen in den ZKPs zu vermitteln sind 5 6 . Dies ist nicht nur für einen präventiven Ansatz hinderlich, sondern kann auch bei den Erziehungsberechtigten zu Verwirrung und mangelnder Kompetenz führen 7 . Daher war es Ziel dieser fragebogengestützten Studie, den Inhalt, die Verständlichkeit und den formalen Aufbau aller in Deutschland verfügbaren ZKPs durch einen validierten Kriterienkatalog zu eruieren, um so fundierte Empfehlungen für die Neugestaltung eines nationalen ZKPs zu geben. Diese Empfehlungen sollen genutzt um nachfolgend anhand des Kriterienkatalogs deutschlandweit einen einheitlichen ZKP für Eltern und Kind zu erstellen, sodass jedes Kind - egal in welchem Kammerbereich befindlich – von der gleichen Qualität an zahnmedizinischen Informationen profitieren kann.

## Methoden

Der Kriterienkatalog zur Bewertung der ZKPs wurde von drei Zahnärzten der Tätigkeitsschwerpunkte in Kinder-, und Jugendzahnmedizin, Parodontologie und ohne Tätigkeitsschwerpunkt erstellt und nach einem Testdurchlauf mit zwei weiteren Zahnmedizinern mit Bestimmung der Intrarater- sowie Interrater-Reliabilität validiert. Dazu wurden alle zu untersuchenden ZKPs (n=14) nacheinander von einem Untersucher bewertet. Die Zweitbewertung erfolgte nach drei Monaten vor Evaluationsbeginn (Erstbewertung 10.09.2021 bis 12.09.21, Zweitbewertung 11.12.21). Alle in Deutschland verfügbaren ZKPs (n=14) wurden für die Auswertung durch die Untersucher in gedruckter sowie digitaler Form ausgehändigt. Jeder Untersucher führte die Bewertung des ZKP für sich bei Univis (Unipark, QuestBack GmbH, Köln, Deutschland) durch.

Insgesamt beinhaltete der validierte Katalog 81 Fragen, eingebettet in 15 Themenkomplexen ( Tab. 1 ), mit welchem ein adäquater inhaltlicher Umfang für die Beantwortung der Fragestellung erzielt werden soll. Dabei beinhaltete der Themenkomplex „Allgemeines“ (Abfrage von: Vorhandensein einer Einleitung, persönliche Dateneingabe des Kindes, altersentsprechende Einteilung des ZKP nach FU-Untersuchungen), der Themenkomplex „Schwangere“ (Empfehlungen zur Zahngesundheit für die werdende Mutter, Kariesrisiko, Parodontalstatus der Mutter), die Themenkomplexe „Anamnese“ und „Befund“ (Kariesrisiko, KFO-Befund, mögliche Habits, etwaiges Stillen), „Zahnärztliche Frühuntersuchung“ (Erwähnung und Empfehlung der FU-Untersuchungen, Erläuterungen der Vorteile, Risiken bei Unterlassen), „Ernährung“ (Zwischenmahlzeiten, Gefahren der Nuckelflasche, Obstsäfte als Risikofaktor, richtiges Timing des Zähneputzens, versteckte Zucker und Säuren) und weiterhin der Themenkomplex „Durchbruchszeiten“. Die Themenkomplexe „Prävention & Mundhygiene“ (Fissurenversiegelung, Zahnputzmethoden, Putzverhalten des Kindes, Mundhygiene-Utensilien) sowie „Fluoride“ (Erwähnung und Empfehlung der Fluoride, Pro- und Contra, lokale und systematische Gabe, Wirkungsweise und Risiken bei Nichtgabe/übermäßiger Gabe, Intensiv- und Basisprophylaxe, Einnahmehinweise, Vorteile einer frühzeitigen Gabe, Fluoridanamnese), „ECC“ (Kariesdefinition, Empfehlungen zur Prävention, Therapiemöglichkeiten), „MIH“ und „Zahnunfall“ schlossen sich an. Abschließend folgten die Themenkomplexe zum formalen Aufbau der ZKPs im Rahmen des „DISCERN“, der „Wittener Liste“ sowie dem „Hamburger Verständlichkeitsmodell“.

**Table:** **Tab. 1** Darstellung der Themenkomplexe des aus 81 Fragen bestehenden Kriterienkatalogs.

Einführung	
Allgemeines	1. TeilnehmerIN 2. Von welchem Bundesland ist dieser Zahnärztliche Kinderpass?
Allgemeines	3. Gibt es eine Einleitung? 4. Können persönliche Daten (Name, Geburtsdatum etc.) des Kindes eingetragen werden, sodass ersichtlich wird, wem der Pass gehört? 5. Gibt es eine altersentsprechende Einteilung des Passes bezüglich der Untersuchungen in z. B. FU1a-c, FUPr, FLA?
Schwangere	6. Werden Empfehlungen zur Zahngesundheit der Mutter in der Schwangerschaft gegeben? 7. Wird das Kariesrisiko und der Parodontalstatus der werdenden Mutter abgefragt?
Anamnese	8. Gibt es eine Anamnese? 9. Hat Ihr Kind eine Erkrankung? 10. Nimmt Ihr Kind Medikamente ein? 11. Hat Ihr Kind Allergien?
Befund	12. Ist ein Feld zum Eintragen des Befunds des Kindes vorhanden? 13. Kann man das Kariesrisiko (z. B. DMFT?) des Kindes eintragen? 14. Kann man einen KFO-Befund des Kindes eintragen? 15. Werden mögliche Habits des Kindes im Befund abgefragt? 16. Wird gefragt, ob und/oder wie lange das Kind gestillt wurde?
Zahnärztliche Frühuntersuchung	17. Wird die Frühuntersuchung erwähnt? 18. Wird die Frühuntersuchung empfohlen? 19. Wird erwähnt, dass bei einer Frühuntersuchung Erkrankungen und Fehlentwicklungen erfasst werden? 20. Wird erwähnt, dass durch die Frühuntersuchung eine Gewöhnung an die zahnärztliche Routine stattfindet? 21. Wird erwähnt, dass durch die Frühuntersuchung eine Entwicklung/ein Bewusstsein für Zahnhygiene entsteht? 22. Wird über die Risiken ohne Frühuntersuchung aufgeklärt?
Ernährung	23. Wird darüber aufgeklärt, dass es besser ist, wenige Zwischenmahlzeiten zu sich zu nehmen? 24. Wird über die „Gefahr“ der Nuckelflasche aufgeklärt? 25. Werden Obstsäfte als mögliches Risiko erwähnt? 26. Wird das richtige Timing des Zähneputzens angesprochen? 27. Werden versteckte Säuren erwähnt? 28. Werden versteckte Zucker erwähnt?
Durchbruchszeiten	29. Werden die Durchbruchszeiten im Milch- und bleibenden Gebiss aufgezeigt?
Komplex Prävention & Mundhygiene	30. Werden Präventionsmaßnahmen angesprochen (Fissurenversiegelung und Prophylaxe)? 31. Werden altersentsprechende Zahnputztechniken empfohlen (z. B. KAI-Methode)? 32. Wird das Zahnputzverhalten des Kindes erfragt (Häufigkeit, Nachputzen)? 33. Werden Utensilien für die Mundhygiene angesprochen: Kinderzahnbürste? 34. Werden Utensilien für die Mundhygiene angesprochen: Zwischenraumbürsten oder Zahnseide?
Fluoride	35. Wird das Wort Fluoridierung erwähnt? 36. Werden Fluoridempfehlungen ausgesprochen (entsprechend der Richtlinien)? 37. Wird ein Standpunkt Pro/Contra bezüglich Fluoriden vertreten? 38. Wird die systematische und die lokale Fluoridierung genauer erklärt? 39. Wird die Wirkungsweise von Fluoriden erläutert? 40. Wird über Risiken bei einer Nichtgabe von Fluoriden aufgeklärt? 41. Wird über Risiken und Folgen bei einer übermäßigen Gabe von Fluorid aufgeklärt? 42. Wird unterteilt in eine Basis- und eine Intensivprophylaxe? 43. Wird die Basis- und die Intensivprophylaxe erläutert? 44. Werden Einnahmehinweise bezüglich Fluoriden gegeben (Menge, Häufigkeit)? 45. Werden die Vorteile einer frühzeitigen Gabe von Fluorid erwähnt? 46. Wird gefragt, welche anderen Fluoridquellen das Kind benutzt (fluoridiertes Salz, Fluoridtabletten etc.)?
ECC	47. Wird erläutert, was eine Karies ist? 48. Werden Empfehlungen zur Prävention der ECC (wie zum Beispiel die Ernährung und das Trinkverhalten) gegeben? 49. Werden Therapiemöglichkeiten zur ECC gegeben?
MIH	50. Gibt es Informationen zu dem Krankheitsbild MIH?
Zahnunfall	51. Wird thematisiert, was bei einem Zahnunfall gemacht werden soll?
DISCERN	52. Sind die Ziele des Passes klar? 53. Bewerten Sie, inwieweit der Pass die selbstgesteckten Ziele erreicht. 54. Ist aus Ihrer Sicht der Pass für die Anwendergruppe (Eltern, Kind) bedeutsam? 55. Existieren klare Angaben zu den Informationsquellen, die zur Erstellung des Passes herangezogen wurden? 56. Ist klar angegeben, wann die Informationen, die in dem Pass verwendet und wiedergegeben wurden, erstellt wurden? 57. Ist der Pass ausgewogen und unbeeinflusst geschrieben worden? 58. Enthält der Pass detaillierte Angaben über ergänzende Hilfen und Informationen? 59. Äußert sich der Pass zu Bereichen, für die keine sicheren Informationen vorliegen? 60. Beschreibt der Pass die Wirkungsweise jedes Behandlungsverfahrens? 61. Beschreibt der Pass den Nutzen jedes Behandlungsverfahrens? 62. Beschreibt der Pass die Risiken jedes Behandlungsverfahrens? 63. Beschreibt der Pass mögliche Folgen einer Nicht-Behandlung? 64. Beschreibt der Pass, wie die Behandlungsverfahren die Lebensqualität beeinflussen? 65. Ist klar dargestellt, dass mehr als ein mögliches Behandlungsverfahren existieren kann? 66. Ist der Pass eine Hilfe für eine „partnerschaftliche Entscheidungsfindung“ (das sogenannte shared-decision-making)? 67. Bewerten Sie abschließend, auf der Grundlage der Antworten, den Pass hinsichtlich der Gesamtqualität als Informationsquelle über Behandlungsalternativen.
Wittener Liste	68. Zielgruppe und Ziel angegeben? 69. Alltagsbezug vorhanden? 70. Positive Bewältigung beabsichtigt? Persönliche Ansprache? 71. Umfang und Schriftgröße? 72. Verständlichkeit? 73. Layout/Überschriften/Abbildungen/Gliederung? 74. Neuzeitliches Wissen/Literaturstützung/Quellen/Datum? 75. Autorenhinweise/Finanzierung/Abhängigkeit? 76. Weiterführende Hinweise/Adressen? 77. Vollständigkeit?
Hamburger Verständlichkeitsmodell	78. Ist der Text einfach geschrieben worden? 79. Ist der Text gegliedert und folgerichtig? 80. Ist der Text kurz und prägnant? 81. Gibt es anregende Zusätze?

### Fragen zum formalen Aufbau und Verständlichkeit

Zur Bewertung des formalen Aufbaus sowie der Verständlichkeit wurden das etablierte DISCERN-Instrument 5 8 , das Hamburger Verständlichkeitsmodell sowie die Wittener Liste 9 verwendet.

Der DISCERN-Fragenkatalog wurde als Instrument zur Bewertung von gedruckten Patienteninformationen eingesetzt, um eine Differenzierung der Qualität (Behandlungsalternativen) durchzuführen. Die Gewichtung der Items erfolgte auf einer Likert-Skala von 1 (Nein), 2–3 (Teilweise), 4–5 (Ja) oder von 1 (Niedrig, beträchtliche Mängel), 2–3 (Mittel, eventuell wichtige, aber nicht beträchtliche Mängel), 4–5 (Hoch, minimale Mängel). Die vor 15 Jahren an der Universität Witten/Herdecke entwickelte Wittener Liste diente ebenfalls der Qualitätssicherung von Patienteninformationen, welche den Fokus mehr auf das Layout sowie etwaige Autorenhinweise und Abhängigkeiten legt. Die Antwortmöglichkeiten entsprechend der Likert-Skala waren von 1 (Trifft nicht zu), 2 (Trifft teilweise nicht zu), 3 (Teils/teils), 4 (Trifft teilweise zu) und 5 (Trifft völlig) möglich. Das Hamburger Verständlichkeitsmodell 10 , welches zur Bewertung der Einfachheit, Gliederung und Prägnanz der Texte entwickelt wurde, legte die Gewichtung der Skalierung der Frageitems auf 1 (− −), 2 (−), 3 (+/−), 4 (+) und 5 (++ ).

### Statistische Analyse

Die statische Auswertung erfolgte nach Ende der Datenerfassung und einem Datenexport aus Univis (Unipark, QuestBack GmbH, Köln, Deutschland) in SPSS Statistics (IBM, Chicago, IL, USA) und beinhaltete neben demographischen Daten der Untersucher (Geschlecht, Untersucherspezialisierung, Untersucherberufserfahrung und ZKP-Kammerbereiche (n=15)) eine deskriptive und statistische Analyse mittels nicht-parametrischer Tests (Mann-Whitney U bzw. Kruskal-Wallis-Test für Mehrfachvergleiche), Spearman-Rho-Korrelationen (Spearman’sches Rho: ρ) sowie einer binären logistischen Regressionsanalyse zum Einfluss der Variablen Spezialisierung des Untersuchers (auf die Zielgruppe/nicht auf die Zielgruppe), Geschlecht der Untersucher (weiblich/männlich), Berufserfahrung der Untersucher (≤7Jahre/>7Jahre) sowie des ZKP-Kammerbereiches (östlicher/ westlicher Kammerbereich) auf den inhaltlichen Summenwert des ZKP (binär codiert als≤Median vs.>Median des Summenwerts aller 81 Items aller Bewertungen aller Untersucher (n=168)). Das festgelegte Signifikanzniveau lag bei p≤0,05, alle statistischen Tests wurden zweiseitig durchgeführt.

## Ergebnisse

### Inhaltlich

**Abb. 1:**
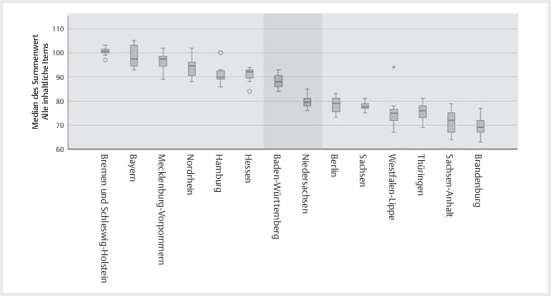
Illustration des Rankings der zahnärztlichen Kinderpässe (ZKP) je Kammerbereich (KB) entsprechend des Summenwertes aller inhaltlicher Items (n=12), wobei sich beim Median des Summenwerts der ZKPs ausgehend von dem am besten bewerteten ZKP aus Bremen und Schleswig-Holstein (identische Ausführung) bis einschließlich Baden-Württemberg keine signifikanten Unterschiede finden (p>0,345). Ebenfalls unterscheiden sich die medianen Summenwerte nicht signifikant aufsteigend vom am unverständlichsten beurteiltem ZKP aus Brandenburg bis Niedersachsen (p>1,00).

Bei Betrachtung des Summenwerts des inhaltlichen Themenkomplexes (n=12 mit 48 Fragenitems; nur die Items des inhaltlichen Komplexes aus dem Kriterienkatalog aufsummiert und entsprechend der Likert-Skala waren maximal 240 Punkte erreichbar) wurde der Kinderpass von Bremen und Schleswig-Holstein (beide Kammerbereiche nutzen einen identischen Kinderpass) am höchsten mit einem Median von (25% / 75% Perzentil) 100,50 (100,00/101,75) bewertet, wohingegen der Kinderpass aus Brandenburg den geringsten Summenwert erreichte 69,00 (66,50/73,00) ( Abb. 1 ).

### Themenkomplex Fluoride

**Abb. 2:**
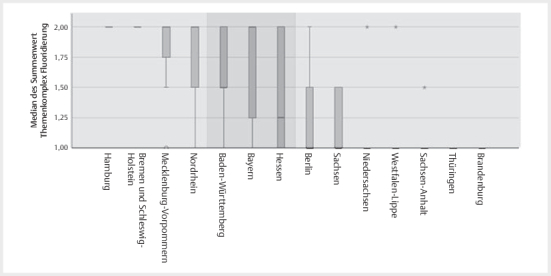
Illustration des Rankings der zahnärztlichen Kinderpässe (ZKP) je Kammerbereich (KB) entsprechend des Themenkomplexes Fluoridierung, wobei sich der Summenwert der ZKPs ausgehend von dem am besten bewerteten ZKP aus Hamburg bis Hessen nicht signifikant unterschied (p>0,445). Ausgehend vom am schwächsten beurteilten ZKP aus Brandenburg aufsteigend bis Baden-Württemberg fanden sich ebenfalls keine signifikanten Unterschiede (p>0,113).

Es fanden sich bezüglich des Fluorid-Komplexes Informationen in nahezu allen ZKPs (n=12). Die Hinweise zur Fluoridierung in den ZKPs der Kammerbereiche Hamburg und Schleswig-Holstein/ Bremen wurden mit 2,00(2,00/2,00) von allen Untersuchern am umfangreichsten bewertet, wohingegen in den ZKPs der Kammerbereiche Thüringen und Brandenburg aus Sicht der Untersucher Angaben zur Fluoridierung ganz fehlten ( Abb. 2 ).

### Themenkomplex Frühuntersuchungen

Die zahnärztlichen FUs wurden laut der Mehrheit der 12 Untersucher bei allen ZKPs thematisiert. So waren sich alle 12 Untersucher bei den Kammerbereichen Bayern, Bremen/ Schleswig-Holstein, Hessen und Hamburg einig, dass die Frühuntersuchungen erwähnt sowie auch empfohlen wurden. Bei den Kammerbereichen Baden-Württemberg, Niedersachsen und Nordrhein waren sich ebenfalls alle 12 Untersucher einig, dass dieser Komplex thematisiert wurde. Hingegen waren nur 7 von 12 Untersuchern der Meinung, dass im ZKP des Kammerbereiches Sachsen-Anhalt dieser Komplex erwähnt sowie empfohlen wurde.

### Themenkomplex ECC, Ernährung sowie Prävention und Mundhygiene

Bei der Betrachtung der Summenwerte im Themenkomplex ECC wies der ZKP des Kammerbereiches Hessen die beste Bewertung mit 2,00(2,00/2,00) auf, Brandenburg mit 1,00(1,00/1,00) dagegen die schlechteste. Im Themenkomplex zur Prävention und Mundhygiene war der ZKP des Kammerbereiches Brandenburg mit 1,00(1,00/1,00) am schwächsten, wohingegen der ZKP des Kammerbereiches Baden-Württemberg mit 2,00(2,00/2,00) am besten bewertet wurde. Für den Themenkomplex der Ernährung wurde der ZKP des Kammerbereiches Niedersachsen mit 2,00(2,00/2,00) am besten, Berlin mit 1,00(1,00/1,00) am schlechtesten bewertet.

### Themenkomplex MIH

Die Molaren-Inzisiven-Hypomineralisation (MIH) wurde für die Mehrheit der ZKPs (Mecklenburg-Vorpommern, Westfalen-Lippe, Thüringen, Hamburg, Niedersachsen, Nordrhein, Sachsen, Brandenburg und Sachsen-Anhalt) nicht benannt. Nur in zwei ZKPs der Kammerbereiche Bremen/ Schleswig-Holstein sowie Baden-Württemberg befanden die Untersucher das Thema MIH erwähnt.

### Themenkomplex Zahntrauma

Hier beurteilten alle Untersucher, dass sich nur in den drei ZKPs der Kammerbereiche Bayern, Thüringen und Sachsen Hinweise zu Zahnunfällen befanden.

### Qualitätskriterien für Patienteninformationen und spezielle Themenkomplexe

Entsprechend der Wittener Liste sowie dem DISCERN Fragebogen wurde der ZKP des Kammerbereiches Hessen von allen Untersuchern mit 5,00(4,13/5,00) / 4,25(4,00/5,00) am besten bewertet, der ZKP des Kammerbereiches Brandenburg mit 2,00(1,63/2,38) / 1,00(1,00/1,00) am geringsten.

**Abb. 3:**
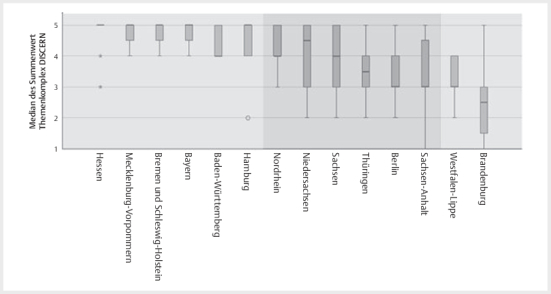
Illustration des Rankings der zahnärztlichen Kinderpässe (ZKP) je Kammerbereich (KB) entsprechend der Frage „Sind die Ziele des Passes klar?“, wobei der Summenwert der ZKPs ausgehend von dem am besten bewerteten ZKP aus Hessen bis einschließlich Sachsen-Anhalt keine statistisch signifikanten Unterschiede zeigte (p>0,214). Die ZKPs aus Brandenburg bis einschließlich Nordrhein unterschieden sich ebenfalls nicht statistisch signifikant (p>0,069).

**Abb. 4:**
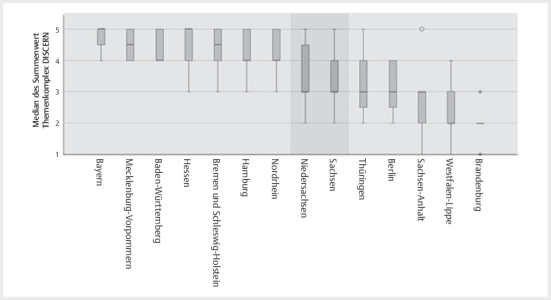
Illustration des Rankings der zahnärztlichen Kinderpässe (ZKP) je Kammerbereich (KB) entsprechend der Frage „Ist aus Ihrer Sicht der Pass für die Anwendergruppe (Eltern Kind) bedeutsam?“, wobei der mediane Summenwert der ZKPs ausgehend von dem am besten bewerteten ZKP bis einschließlich Sachsen statistisch nicht signifikant war (p>0,084). Ebenfalls unterschieden sich die medianen Summenwerte ausgehend vom ZKP aus Brandenburg bis einschließlich Niedersachsen statistisch nicht signifikant (p>0,260).

Eine Auswertung von einzelnen Items des DISCERN Fragebogens ob (a) die Ziele der ZKPs klar definiert sind ( Abb. 3 ) und (b) die Untersucher den Inhalt des Kinderpasse als bedeutsam für die Anwendergruppe bewerteten ( Abb. 4 ), ergab, dass der ZKP des Kammerbereiches Hessen mit 5,00 (5,00/5,00) am besten die Ziele des ZKPs erreicht und Brandenburg mit 2,50(1,25/3,00) am wenigsten. Der ZKP des Kammerbereiches Bayern wurde für die Anwendergruppe am bedeutsamsten 5,00(4,25/5,00) bewertet, wohingegen erneut der ZKP des Kammerbereiches Brandenburg mit 2,00(2,00/2,00) die geringste Bewertung aufwies.

### Hamburger Verständlichkeitsmodell

Der höchste Summenwert des Hamburger Verständlichkeitsmodells fand sich für den ZKP des zahnärztlichen Kammerbereiches Bayern mit einem Median von 4,00(4,00/4,88), wohingegen Brandenburg mit 2,75(2,13/3,50) als am wenigsten verständlich beurteilt wurde. Je inhaltlich gehaltvoller ein ZKP war, umso verständlicher empfanden ihn auch die Untersucher (ρ=0,449 (p<0,001)).

### Einflussfaktoren der Untersuchung

Die abschließende binäre logistische Regressionsanalyse zum Einfluss von vier Untersuchungsparameter auf die inhaltliche Gesamtqualität identifizierte nur die Variable Kammerbereich mit einer 15-fachen Zunahme der inhaltlichen Gesamtqualität für die westlichen ZKP-Kammerbereiche als signifikant (p<0,001) ( Tab. 2 ).

**Table:** **Tab. 2** Ergebnisse der Regressionsanalyse zum Einfluss von vier verschiedenen Variablen (Spezialisierung, Geschlecht und Berufserfahrung der Untersucher, Kammerbereich Ost vs. West) auf die Inhaltliche Gesamtqualität der kinderzahnärztlichen Pässe (B: Regressionskoeffizient; SE: Standardfehler des Regressionskoeffizienten B; p-Wert: Signifikanzwert; OR: Odds Ratio).

Variable	B	SE	OR	95% Konfidenzintervall		*p-* Wert*
				untere Grenze	obere Grenze	
**Spezialisierung (Referenz: nicht auf die Zielgruppe spezialisiert)**	−0,018	0,482	0,982	0,382	2,527	0,970
**Geschlecht (Referenz: weiblich)**	0,144	0,537	1,155	0,403	3,312	0,788
**Berufserfahrung (Referenz:<7Jahre)**	0,269	0,403	1,309	0,594	2,881	0,504
**Kammerbereiche (Referenz: OST)**	2,722	0,397	15,210	6,991	33,093	<0,001
**Konstante**	−1,779	0,403	0,169			<0,001

## Diskussion

Ein Zahnärztlicher Kinderpass (ZKP) kann helfen, Eltern und Kinder besser über die Mundgesundheit zu informieren und zu motivieren sowie präventive Maßnahmen einschließlich Vorsorgetermine in der zahnärztlichen Praxis wahrzunehmen. Oftmals bereitet es den Eltern große Schwierigkeiten für eine adäquate Zahngesundheit ihrer Kinder ohne Anleitung zu sorgen 5 . Es kann festgestellt werden, dass die Studienergebnisse neben den vielen Stärken einzelner ZKPs insgesamt auch deutliche Qualitätsdiskrepanzen von ZKPs aufweisen. Insbesondere schnitten Schleswig-Holstein, Bremen, Bayern, Nordrhein, Hamburg, Hessen sowie Baden-Württemberg mit einem vergleichbaren Inhalt besser ab als Berlin, Sachsen, Thüringen, Sachsen-Anhalt sowie Brandenburg.

### Fluoridierung

Beispielsweise beurteilten 58,3% aller Untersucher bei den ZKPs der Kammerbereiche Westfalen-Lippe, Berlin und Sachsen, dass aktuelle Empfehlungen zur Fluoridierung 11 nicht erwähnt wurden, bei Thüringen und Brandenburg waren es 66,7% und bei Sachsen-Anhalt sogar 75,0% aller Untersucher. Dabei ist gerade das Thema der Fluoridierung von herausragender Bedeutung, da diese als Hauptursache für den Rückgang der Kariesprävalenz in Deutschland angesehen wird 12 . So konnte gezeigt werden, dass eine 1000–1450 ppm hohe Fluoriddosis zu einer Kariesreduktion um 22% führen kann und eine positive Korrelation zwischen der Konzentration des Fluorids sowie seiner Wirkung in der Kariesprophylaxe herrscht 12 . Trotzdem besteht immer noch eine von Ängsten und Fehlinformationen getriggerte Diskussion um das Nutzen-Risiko-Verhältnis der Fluoridierung 5 . Beispielweise wussten 2020 viele Eltern nicht, wie viel Fluorid eine Zahnpasta enthalten sollte 13 . Somit ist die Illustration der empfohlenen reiskorngroßen Fluoridmenge in einzelnen ZKPs (z. B. von Schleswig-Holstein/Bremen) hilfreich, die richtige Dosierung einzuhalten, da oftmals überdosiert wird 14 .

### Vorsorge

Bedingt durch die Studienmethode können keine Aussagen zur Inanspruchnahme der zahnärztlichen Präventionsmaßnahmen getroffen werden und es war nicht vorgesehen, spezifische Interventionen der gesetzlichen Krankenversicherung (GKV) zu hinterfragen oder zu analysieren ebenso wenig wie die Koordination der unterschiedlichen zahnärztlichen Präventionsmaßnahmen nicht Gegenstand der Analyse war.

Bei den ZKPs der Kammerbereiche Bayern, Bremen/ Schleswig-Holstein, Hessen, Hamburg, Baden-Württemberg, Niedersachsen, Nordrhein und Sachsen waren sich 100% der Untersucher einig, dass die FU erwähnt wurden, beim ZKP aus Sachsen-Anhalt allerdings nur 58,3%. Alle Untersucher waren sich einig, dass die FUs in den ZKPs der Kammerbereiche Bayern, Bremen/Schleswig-Holstein, Hessen, Mecklenburg-Vorpommern und Hamburg empfohlen wurden. Geiken et al. fanden im Rahmen einer fragebogengestützten Onlinestudie heraus, dass 35,5% der befragten 696 deutschen Kinderärzte keinerlei FUs bei (Kinder-)Zahnärzten vorsehen und 20,6% der Befragten Kinder unter einem Jahr für zu jung für solch eine FU hielten, obwohl die derzeitigen nationalen Empfehlungen einen ersten Besuch des Kindes mit 6 Monaten vorsehen 15 . Dennoch, auch die Mehrheit der befragten Kinderärzte gaben 2022 an, ab dem ersten Milchzahn eine Überweisung zum Kinderzahnarzt für sinnvoll zu erachten 4 . Auch ist zu konstatieren, dass Kinder, welche vor dem zweiten Lebensjahr eine FU in Anspruch genommen haben, weniger proaktiv behandelt werden mussten, als nach dem zweiten Lebensjahr 16 . Die ZKPs können dabei helfen, sowohl an die FUs zu erinnern als auch als eine Art Checkliste für die Eltern zu dienen, wann und welche FU-Interventionen noch bevorstehen.

### MIH und Zahntraumata

Bemerkenswert in der vorliegenden Studie stellte sich der Themenkomplex der MIH dar, welcher bei der Mehrheit der ZKPs (Mecklenburg-Vorpommern, Westfalen-Lippe, Thüringen, Hamburg, Niedersachsen, Nordrhein, Sachsen, Brandenburg und Sachsen-Anhalt) gar nicht benannt wurde. Zwar findet sich nur eine globale Prävalenz von 14,2% für MIH 17 , aber in letzter Zeit wurde das Thema der „Kreidezähne“ als potentieller Angstauslöser bei Eltern und Kinder medienwirksam präsentiert. Ein erhöhtes Maß an Hypersensibilität und Kariesrisiko führt unbehandelt oftmals zu einer signifikanten Verschlechterung der Zahngesundheit des betroffenen Kindes 18 , weshalb eine frühe Diagnose von herausragender Bedeutung ist. Auch beim Themenkomplex der Zahntraumata beurteilten alle Untersucher dieser Studie, dass sich Informationen hierzu nur in den drei ZKPs der Kammerbereiche Bayern, Thüringen und Sachsen fanden. Das mangelnde Fachwissen der Eltern kann die Prognose eines solchen Zahnes drastisch verringern 19 . Eine im Jahre 2013 veröffentlichte Studie aus dem Iran zeigte, dass eine Broschüre mit Informationen bezüglich Zahntraumata dabei maßgeblich helfen konnte, dass Eltern im Falle eines Zahntraumas adäquater handeln und somit die Prognose von avulsierten Zähnen sublimiert wird 20 .

### Ost-West-Analyse der Kammerbereiche

Die Ergebnisse der Ost-West-Analyse der Kammerbereiche weisen eine eingeschränkte Anwendbarkeit auf und sollten daher mit Umsicht interpretiert werden und nicht als alleiniger Maßstab zur Bewertung der Inhalte herangezogen werden! Zwar erzielten die westlichen Kammerbereiche (Bayern, Schleswig-Holstein, Bremen, Westfalen-Lippe, Hamburg, Baden-Württemberg, Niedersachsen und Nordrhein-Westfalen) im Vergleich zu den östlichen Kammerbereichen (Berlin, Brandenburg, Sachsen, Sachsen-Anhalt, Thüringen) mehrheitlich bessere Ergebnisse, doch sollte dies nicht in einer pauschalen und teils emotional geführten Diskussion von „Ost versus West“ verallgemeinert werden. Beispielsweise zeigte die Analyse unserer Ergebnisse, dass während viele östliche Kammerbereiche am unteren Ende des Rankings liegen, der Kammerbereich Mecklenburg-Vorpommern, tendenziell sehr gute Ergebnisse aufweist. Umgekehrt sind die durchweg positiven Ergebnisse der westlichen Kammerbereiche nicht auf den Kammerbereich Westfalen-Lippe zutreffend. Wie die Daten der letzten deutschen Mundgesundheitsstudien zeigten, haben sich die einst deutlichen Unterschiede in der oralen Gesundheit zwischen den östlichen und westlichen Bundesländern stark in den vergangenen Jahrzehnten stark nivelliert 21 22 23 . Wir bitten deshalb die Leser, die vorliegenden Ergebnisse differenziert und umsichtig zu interpretieren.

### Verständlichkeit und Quellenhinweise

Es fanden sich nicht nur inhaltliche Diskrepanzen, sondern auch welche in der Verständlichkeit der ZKPs. Entsprechend der Wittener Liste und des DISCERN bewerteten die Untersucher den ZKP des Kammerbereiches Hessen am verständlichsten, den des Kammerbereiches Brandenburg am unverständlichsten. Dies kann bedeuten, dass beispielsweise eine Erwähnung von Behandlungsalternativen sowie Risiken im Sinne der partizipativen Entscheidungsfindung (engl.: shared-decision-making; kurz SMD) mit unverständlicheren ZKPs kaum möglich ist 24 . So zeigt der 2024 veröffentlichte HTA-Bericht (Health Technology Assessment) des Instituts für Qualität und Wirtschaftlichkeit im Gesundheitswesen (IQWiG), dass vor allem Entscheidungshilfen die Kommunikation und das Wissen der Patienten verbesserten. Diese Entscheidungshilfen stellen sich in Form von Informationsmaterialien dar, welche eine Pro- und Contra-Seite aufzeigen 23 .

## Zusammenfassung

Insgesamt können die vorliegenden Erkenntnisse der Studie durch den erstmals erstellten Kriterienkatalog Empfehlungen zur Gestaltung eines idealen zahnärztlichen Kinderpasses geben und sollten als Basis genutzt werden, um zukünftig einheitliche, fundierte Informationen und Empfehlungen zu geben, sodass der Hauptindikator für die Qualität und Quantität der zahnärztlichen Kinderpässe nicht mehr der Parameter des veröffentlichenden zahnärztlichen Kammerbereichs ist.
